# Systemic Glucose Administration Alters Water Diffusion and Microvascular Blood Flow in Mouse Hypothalamic Nuclei – An fMRI Study

**DOI:** 10.3389/fnins.2019.00921

**Published:** 2019-09-03

**Authors:** Blanca Lizarbe, Antonio Fernández-Pérez, Victor Caz, Carlota Largo, Mario Vallejo, Pilar López-Larrubia, Sebastián Cerdán

**Affiliations:** ^1^Instituto de Investigaciones Biomédicas “Alberto Sols” CSIC-UAM, Madrid, Spain; ^2^Centro de Investigación Biomédica en Red de Diabetes y Enfermedades Metabólicas Asociadas (Ciberdem), Instituto de Salud Carlos III, Madrid, Spain; ^3^Departamento de Cirugía Experimental, Instituto de Investigación Hospital Universitario La Paz – IdiPAZ, Madrid, Spain

**Keywords:** glucose, hypothalamus, magnetic resonance imaging, energy metabolism, diabetes

## Abstract

The hypothalamus is the principal regulator of global energy balance, enclosing additionally essential neuronal centers for glucose-sensing and osmoregulation. Disturbances in these tightly regulated neuronal networks are thought to underlie the development of severe pandemic syndromes, including obesity and diabetes. In this work, we investigate *in vivo* the response of individual hypothalamic nuclei to the i.p. administration of glucose or vehicle solutions, using two groups of adult male C57BL6/J fasted mice and a combination of non-invasive T_2_^∗^-weighted and diffusion-weighted functional magnetic resonance imaging (fMRI) approaches. MRI parameters were assessed in both groups of animals before, during and in a *post-stimulus* phase, following the administration of glucose or vehicle solutions. Hypothalamic nuclei depicted different patterns of activation characterized by: (i) generalized glucose-induced increases of neuronal activation and perfusion-markers in the lateral hypothalamus, arcuate and dorsomedial nuclei, (ii) cellular shrinking events and decreases in microvascular blood flow in the dorsomedial, ventromedial and lateral hypothalamus, following the administration of vehicle solutions and (iii) increased neuronal activity markers and decreased microperfusion parameters in the ARC nuclei of vehicle-administered animals. Immunohistochemical studies performed after the *post-stimulus* phase confirmed the presence of *c-Fos* immunoreactive neurons in the arcuate nucleus (ARC) from both animal groups, with significantly higher numbers in the glucose-treated animals. Together, our results reveal that fMRI methods are able to detect *in vivo* diverse patterns of glucose or vehicle-induced effects in the different hypothalamic nuclei.

## Introduction

The regulation of glucose homeostasis is an important function involving a delicate interplay between the glucose-sensing mechanisms of the brain and the pancreatic islets, and requires the participation of target organs such as muscles, the adipose tissue and the liver. Both the pancreatic β cells and some cerebral structures have the ability to sense blood glucose levels and convert the information into adaptive signals (Bentsen et al., 2019). In particular, glucose-sensing neurons in the hypothalamus are known to have the capacity to either increase (glucose-excited, GE) or decrease (glucose-inhibited, GI) their electrical activity by detecting fluctuations in extracellular glucose levels (Fioramonti et al., 2017; Stanley et al., 2019). These glucose sensing-neurons are strategically located within several interconnected hypothalamic nuclei that play key roles in the regulation of the mechanisms maintaining global energy homeostasis. In the arcuate nucleus (ARC), glucose-sensing neurons are known to modulate the activity of orexigenic Agouti Related Protein/Neuropeptide Y or anorexigenic Pro-opiomelanocortin/Cocaine Amphetamine Related Transcript neurons, respectively (Fioramonti et al., 2007; Belgardt et al., 2009; Kong et al., 2010). On the other hand, some GABA-ergic neurons in the dorsomedial nucleus (DMN) have been reported to be GE- or GI-responsive (Otgon-Uul et al., 2016), while both types of neurons have been equally described in the ventromedial nucleus (VMN) (Toda et al., 2016). In the lateral hypothalamus (LH), orexin-, neuropeptide Y- and GABA- expressing neurons present GI properties, while melanin-concentrating-hormone neurons are GE (Fioramonti et al., 2017). Further coordination between the ARC and the LH play a key role in the brain osmoregulatory mechanisms, establishing a pathway that links nutrient and fluid balance (van Dijk et al., 2011). Importantly, disruptions in the balance of these delicate mechanisms are believed to underlie diseases with enormous morbidity and mortality worldwide, including obesity and diabetes (Marty et al., 2007; Parton et al., 2007; McCrimmon, 2008).

Despite the important progress in the understanding of the different molecular and cellular mechanisms underlying cerebral glucose-sensing and global energy homeostasis (Meek et al., 2016; Soty et al., 2017), their integrative operation and coordinated responses *in vivo* to maintain euglycemia and ensure an efficient energy balance, have remained more difficult to assess. The use of non-invasive evaluation methods has improved considerably our current understanding of hypothalamic function (Lizarbe et al., 2013a). Both Blood Oxygen Level-Dependent (BOLD) functional Magnetic Resonance Imaging (fMRI) and Position Emitted Tomography (PET) approaches have been implemented previously to investigate hypothalamic responses to the administration of glucose (Liu et al., 2000; Mahankali et al., 2000; Smeets et al., 2005; Osada et al., 2017). However, the spatial resolution of both methods and the complex origin of the BOLD signal (Moraschi et al., 2012) limits the applications requiring high spatial and temporal sub-hypothalamic nuclei resolution. To improve these approaches, we proposed recently the use of Diffusion fMRI (DfMRI) as a new methodology to evaluate hypothalamic function in animals and humans (Lizarbe et al., 2013b, 2015; Benitez et al., 2019). This technique is endowed inherently with higher spatial and temporal resolution than BOLD or PET, allowing adequate spatial resolution *in vivo* of the ARC, the VMN, DMN and the lateral hypothalamus. Briefly, DfMRI identifies non-invasively regional cerebral activity by detecting changes in water diffusion occurring in response to activation of specific areas. DfMRI can be selectively sensitized to detect osmotic water redistributions associated to transcellular ionic trafficking, as imposed by propagating action potentials (when using *high b* values), or to assess the intravoxel incoherent motion (IVIM) effects associated with the microvascular perfusion responses to activation (when using *low b* values) (Le Bihan et al., 1986, 2006; Le Bihan, 2008), approximated as pseudo-diffusion processes.

In this work, we aimed to evaluate the integrative response of the hypothalamus to glucose or vehicle administrations *in vivo*, resolving the contributions of oxygenation/hemodynamic or water diffusion or changes in the different sub-hypothalamic nuclei. To this end, we implemented DfMRI and T_2_^∗^-weighted (T_2_^∗^W) MRI on the brain of fasted mice before, during and after the i.p. administration of glucose or vehicle solutions. Immunohistochemical tests of *c-Fos* activation (Wu et al., 2014) were additionally performed to confirm the MRI findings.

## Materials and Methods

### Animals and Experimental Design

All animal procedures were approved by the highest institutional ethical committee (Community of Madrid) and met the Spanish (R.D. 53/2013) and European Community (2010/62/UE) regulations. Mice were housed in cages containing three or four animals per cage, under controlled temperature (21–23^∘^C) and humidity (47%) conditions and 12 h light/dark cycles (8 a.m., lights on). Adult male C57BL6/J mice (Charles River, FR, *n* = 22, 8–11 weeks, 24 ± 1.3 g), individually identified with ear punches, were fasted overnight and imaged before and after the i.p. administration of a solution of glucose (8 mL/kg, 20 mmol/kg) in saline, or only the saline solution (0.9% NaCl, 8 mL/kg, 1.23 mmol/kg) used as vehicle. The administration of glucose or vehicle solutions was performed while mice were in the scanner, using a homemade catheter specially built for this purpose. Animals were divided in four experimental groups: DfMRI/glucose administration (*n* = 7), T_2_^∗^W/glucose administration, (*n* = 7), DfMRI/vehicle administration (*n* = 4) and T_2_^∗^W/vehicle administration (*n* = 4).

**FIGURE 1:**
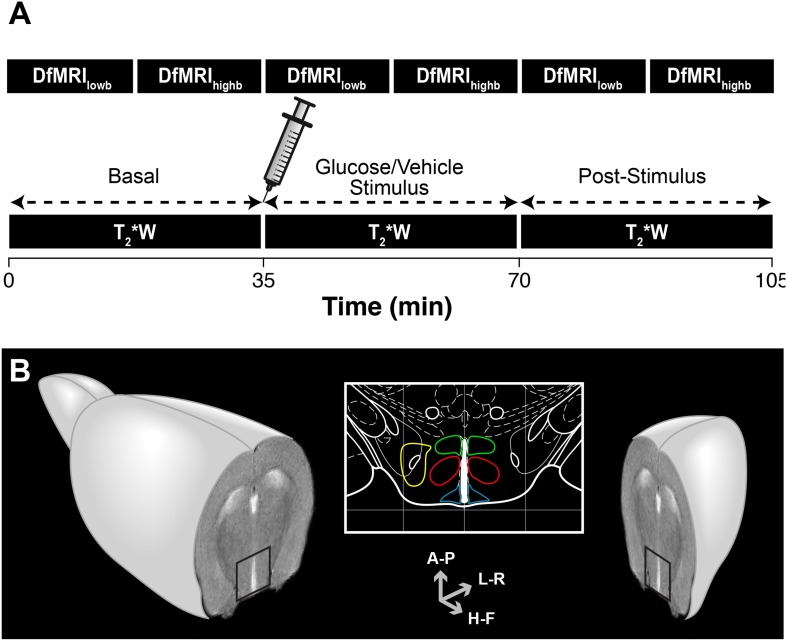
Overview of the methodology implemented in the functional magnetic resonance imaging (fMRI) evaluation of hypothalamic glucose sensing. **(A)** Schematic representation of the MRI protocol. C57BL6/J mice were imaged using either *high b* or *low b* DfMRI or T_2_^∗^W methods. Serial DfMRI or T_2_^∗^W acquisitions were divided into *basal* (starting 35 min before the i.p. solution administration), *Stimulus* (up to 35 min following the i.p. injections) or *post-stimulus*, (starting 35 min. after the i.p. injections). **(B)** Axial MRI section of an idealized mouse brain containing the hypothalamus (black square). The hypothalamic nuclei are depicted in the mouse brain atlas insert: LH (yellow), VMN (red), DMN (green) and ARC (blue). Antero-Posterior (A-P), Left-Right (L-R) and Head-Foot (H-F) orientations of the diffusion gradient.

A general overview of the methodology implemented is depicted in Figure 1A. The DfMRI protocol consisted in acquiring two consecutive DfMRI sequences, first a *low b sequence* and subsequently a *high b acquisition*, before the vehicle or glucose administrations (*basal*), immediately after the i.p. bolus injection (*stimulus*) and 35 min later (*post-stimulus*). The total acquisition time was 105 min. The T_2_^∗^W protocol comprised T_2_^∗^W sequences of identical total duration to the DfMRI protocol, acquired under the *basal*, *stimulus* and *post-stimulus* conditions. All imaging experiments started at 9 a.m. after the overnight fasting. Briefly, two animals from different groups were randomly selected and examined per morning, to minimize uncertainties derived from significantly different durations of the circadian rhythms. This procedure was reproduced until all animals and groups were investigated. During image acquisition protocols, animals were anesthetized with 1–1.5% isoflurane/oxygen, maintaining constant the temperature of the animal at 37^∘^C with a recirculating water blanket. The physiological state of the animal during the imaging process was monitored by the respiratory rate and body temperature using an ECG/Temperature device (Model 1025, Small Animal Monitoring and Gating System, SA Instruments, Inc., NY). Animals were weighted the night before fasting and before the imaging protocols. No adverse effects of these procedures were observed and no animals were excluded from the study. Results are reported in compliance with the ARRIVE (Animal Research: Reporting *in vivo* Research) guidelines.

### Blood Glucose and Hormonal Profiles

After the imaging sessions, blood glucose levels in all animals were measured with a glucometer (One touch Ultra, Lifescan, Johnson and Johnson, Issy-les-Moulineaux, FR) in blood samples drained from the tail vein. Immediately after, blood samples from glucose-administered (*n* = 7) and vehicle-administered (*n* = 4) mice were extracted from the heart and stored for the subsequent analyses of ghrelin, insulin, leptin, glucagon and peptide YY (PYY), using a Mouse Metabolic Hormone Magnetic Bead Panel (Millipore MMHMAG-44K^1^). For each animal, a total of 0.8 ± 0.1 mL of blood was extracted from the heart while the animals were anesthetized with 2% isoflurane/oxygen. All mice were sacrificed after the blood extractions. Experimental protocols for the sample collection, storage, preparation of reagents for immunoassay and immunoassay procedure, followed the specific instructions of the MMHMAG-44K mouse panel supplier. The immunoassay procedure was carried out by the Experimental Surgery Department of the Hospital La Paz Health Research Institute in Madrid, using the Luminex system (Luminex 200^TM^ xPONENT^®^ 3.1 System with MILLIPLEX^®^ Analyst 5.1, Millipore, see text footnote 1).

### fMRI

All images were acquired with a 7T Bruker AVANCE III horizontal magnet (16 cm bore), equipped with a ^1^H selective birdcage resonator (23 mm) located in the center of a 90 mm diameter gradient insert (360 mT/m). Imaging data were acquired using a Linux based Hewlett-Packard console running Paravision 5.1 software (Bruker Medical GmbH, Ettlingen, Germany). Field homogeneity was achieved using the standard methods provided by Bruker in Paravision v.5.1. Briefly, the measurement principle is the observation of the FID-area of a pulse and acquire signal optimized progressively with different shim settings. A user-defined group of shims (7) is implemented in an iterative cycle; each successive shim being adjusted individually to maximize the area of the FID signals in time domain.

The T_2_^∗^W protocol consisted in consecutive gradient-echo FLASH sequences (TR/TE = 182/4 ms, 4 averages) of 80 s duration each, during the 35 min intervals of the *basal*, *stimulus* and *post-stimulus* periods, resulting then in eighteen uninterrupted T_2_^∗^W image acquisitions. DfMRI protocols involved serial consecutive multi-shot diffusion tensor imaging echo-planar acquisitions (DTI EPI) of an axial slice containing the hypothalamus (Figure 1B), under *high b* and *low b* diffusion weightings, in three orthogonal directions; Head-Foot (H-F), Left-Right (L-R) and Antero-Posterior (A-P). *High b* images were acquired using the following parameters; TR/TE = 3000/31 ms, 4 segments, duration of the diffusion gradients δ = 4 ms, separation between the diffusion gradients Δ = 20 ms, 9 *b*-values (300, 400, 600, 900, 1200, 1500, 1600, 1800, and 2000 s/mm^2^), with *b* = γ^2^δ^2^*G*^2^(Δ−δ/3), where γ is the gyromagnetic ratio of the proton and *G* the gradient strength, and 3 averages. *Low b* images were acquired under similar acquisition conditions, orientations and diffusion times, but using nine low *b*-values (10, 30, 60, 100, 150, 200, 300, 400, and 600 s/mm^2^) and number of averages = 2. In all protocols, in-plane resolution and voxel height were identical (0.165 × 0.165 mm^2^ and, 1.125 mm, respectively).

### Image Analysis

Hypothalamic nuclei were identified superimposing the MR images over a mouse brain atlas (Paxinos and Franklin, 2001). Figure 1B shows the anatomical localization of the nuclei investigated: the ARC nucleus (blue, 10 voxels); the VMN (red, 18 voxels); the DMN (green, 12 voxels) and the LH (yellow, 12 voxels). Exceptionally, and due to slight particular anatomical or positional differences of the slice containing the hypothalamus, some the nuclei were selected with ±1 number of voxels.

Signal intensity (SI) of the T_2_^∗^W images from the *stimulus* and *post-stimulus* states was normalized to values from the *basal* state in a voxel-by-voxel manner. Briefly, prior to normalization, the 18 scans-per-condition were subdivided in three consecutive subsets of 6 scans, and outliers within each subset were removed. Outliers included values higher than 1.5 times the inter quartile range + third quartile, or values lower than the first quartile – 1.5 times the inter quartile range. Next, and without outliers, average *basal*, *stimulus* and *post-stimulus* values were calculated for every voxel and normalized to baseline. The coefficient SI/SI_basal_ was assessed for the *stimulus* and *post-stimulus* states and its variation evaluated statistically. The signal-to-noise ratio (SNR) of the T_2_^∗^W images increased after the i.p. administration of the bolus (SNR = 24 ± 3, 30 ± 8, 32 ± 5 a.u. in the *basal*, *stimulus* and *post-stimulus* periods).

Datasets from *high b* sequences were analyzed using either monoexponential (Eq. 1) or biexponential models of diffusion (Eq. 2).

(1) $$S\,\left(\text{b}\right)/S\,\left(0\right)={\text{e}}^{-\text{b}\,\text{ADC}}$$

(2) $$S\,\left(\text{b}\right)/S\,\left(0\right)=\text{SDP}\,{\text{e}}^{-{\text{bD}}_{\text{slow}}}+\text{FDP}\,{\text{e}}^{-{\text{bD}}_{\text{fast}}}$$

Where *S*(*b*) and *S*(*0*) represent the individual voxel intensities in the presence and absence of the diffusion gradients, *b* indicates the diffusion weighting factor (s mm^–2^) and *ADC* the Apparent Diffusion Coefficient. *SDP* and *FDP* represent the slow and fast diffusion phases, respectively (with *SDP* + *FDP* = 1), and *D*_slow_ and *D*_fast_ the corresponding slow and fast diffusion coefficients. Parameters were determined independently for each voxel and direction, using the non-linear least-squares fitting Trust-Region algorithm implemented in Matlab (Matlab R2010b, The MathWorks, Nattick, MA, United States), customized to limit parameter ranges and optimize the goodness of fit. *D*_slow_ and *D*_fast_ coefficients from the biexponential model were fitted for the *basal* condition at a voxel level, and used as fixed parameters for the subsequent conditions (Le Bihan et al., 2006).

*Low b* analysis was performed with a two-step approach (Maximov and Vellmer, 2019). First, and using data from the *basal* condition with 200 < *b-values* < 600 s mm^–2^, a monoexponential equation (Eq. 1) was used to fit a *basal* diffusion coefficient *D* for every voxel and direction. Then, the resulting *D* values were used to fit a biexponential model (Eq. 3) with the whole *low b*-range in the three conditions.

(3) $$S\,\left(\text{b}\right)/S\,\left(0\right)=f\,{\text{e}}^{-\text{bD}}+f^{*}\,{\text{e}}^{-\text{b}\,\left(\text{D}+{\text{D}}^{*}\right)}$$

With *S*(*b*) and *S*(*0*) representing the voxel intensities, *b* the diffusion weighting factor (s mm^–2^), *D* the diffusion coefficient obtained without the 200 < *b-values* < 600 s mm^–2^data, *f* and *f*^∗^ the fraction of diffusing and pseudo-diffusing (or perfusing) molecules (with *f* + *f^∗^* = 1), respectively, and *D*^∗^ the pseudo-diffusion coefficient. The primary monoexponential fit was performed using the non-linear least-squares fitting Trust-Region algorithm described above, and *f*, *f*^∗^ and *D*^∗^ were fitted using a constrained optimization based on the interior-point algorithm (Byrd et al., 2000). SNR of all diffusion-based acquisitions decreased with increasing *b* values, with a maximum at *b* = 0 (SNR = 47 ± 7) and a minimum at *b* = 2000 s/mm^2^ (SNR = 14.2 ± 0.4) for the whole hypothalamic region.

### Immunohistochemistry

Four mice were injected i.p. with glucose (3 g/kg, *n* = 2) or vehicle (0.9% NaCl, *n* = 2) solutions, anaesthetized with 2% isoflurane/oxygen and fixed by transcardial perfusion with 4% paraformaldehyde in phosphate buffered saline (PBS), 1 h after the i.p. injection. Brains were removed, post-fixed in 4% paraformaldehyde (4°C overnight), washed with PBS, and cryoprotected in PBS containing 20% sucrose at 4°C for at least 24 h. Serial coronal sections across the hypothalamic region (20 μm slice thickness) were cut in a Leica CM1950 cryostat and kept at –80°C until used. Sections were thawed, permeabilized with cold methanol for 2 min, treated with 5% normal goat serum, and then incubated overnight at room temperature with a rabbit antiserum against *c-Fos* (PC38, Calbiochem) (1:15000 dilution). Immunodetection was carried out with a secondary biotinylated goat anti-rabbit antiserum (Vector Laboratories; 1:500 dilutions) using diaminobenzidine and nickel-intensified immunoperoxidase staining. Digital images of immunolabeled sections were taken using a Zeiss Axiophot microscope (Wu et al., 2014). Positive nuclei in the region corresponding to the ARC were manually counted using NIH ImageJ software.

### Statistics

Changes in the physiologic measurements were assessed using GraphPad Prism, version 6 (La Jolla, CA). Body weight differences after the overnight fasting were assessed independently for each group with a two-tailed paired *t*-test, and differences in the blood parameters between cohorts were studied either with two-tailed unpaired *t*-tests (glucose comparison after MRI) or using two-way ANOVA followed by independent comparisons with the Fisher’s least significant difference (LSD) test (hormones). Values from the blood tests are presented in bar graphs as mean ± SEM.

Statistical analyses of the MRI parameters were performed with the IBM SPSS package (IBM SPSS Statistics for Windows, Version 25.0, Armonk, NY: IBM Corp.). Parameters from the monoexponential *high b*, biexponential *high b*, *low b* and T_2_^∗^W approaches were independently analyzed using Generalized Estimated Equations (GEE). Briefly, in a GEE approach, an *ad hoc* model is defined in which dependent variables (ADC, SDP, *f*, *D*^∗^ for each direction and SI/SI_basal_) are considered to be linearly related to the independent factors (*basal*, *stimulus* or *post-stimulus* conditions) via a specific link-function. Besides, GEE procedure allows for the analysis of repeated measures (different hypothalamic nuclei and voxels). Once the model is defined, estimated values are calculated, and the effect of the independent factors on the estimated values is subsequently tested statically. Our model was designed as follows: for each model, each voxel was labeled with the information corresponding to the *subject* (identification of the individual mice), type of *stimulus* (0 for vehicle and 1 for glucose), *condition* (0 for *basal*, 1 *stimulus* and 2 for *post-stimulus*), *nuclei* (1 to 4, for ARC, DMN, VMN or LH, respectively) and coordinates (*x*,*y*). A log function was used as link between data and factors, based on a Tweedie distribution correcting for non-normality of the experimental values. Data from the vehicle or glucose groups were assessed separately. *Condition*, *nuclei* and the product *condition*^∗^*nuclei* were used as main predictors for each mice cohort and technique, and significance was tested using a Wald test with a 95% confidence interval. Only values with significance < 0.05 were considered to be statistically different. Values of the parameters that changed significantly between conditions are illustrated in descriptive line graphs, generated using GraphPath, as mean coefficients (per nuclei and condition) ± representative SEM values. These SEM were calculated as the intra-nucleus SD for all animals’ voxels divided by the number of animals investigated. The previously SPSS-calculated statistical significance is shown superimposed to the corresponding line graphs.

## Results

### Physiological Variables

**FIGURE 2:**
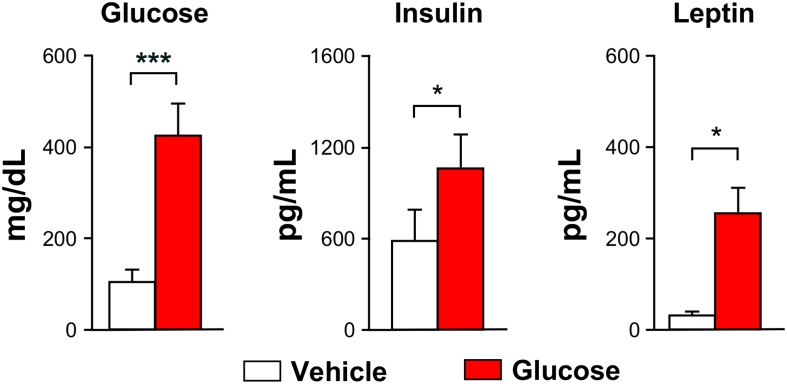
Blood glucose and hormones. Graph bars depicting mean (±SEM) values for the vehicle (white) or glucose (red) administered animals. All values were measured after the MRI evaluations (^∗^*p* < 0.05, ^∗∗∗^*p* < 0.001).

Body weights of the animals undergoing glucose (*n* = 14) or vehicle (*n* = 7) administrations decreased significantly by 11 ± 2% or 11 ± 1%, respectively, during the overnight-fasting period. Blood glucose was significantly higher in the animals receiving glucose administrations, as compared to those receiving saline, measured 70 min after the administrations (Figure 2, left). Both plasma insulin and leptin levels were higher in the glucose cohort, remarkably in insulin (Figure 2, center), while no relevant changes were observed in the plasma ghrelin, glucagon or PYY content between glucose and vehicle groups. Rectal-temperature measurements did not show either significant difference between the different animal groups.

### T_2_^∗^W Imaging

**FIGURE 3:**
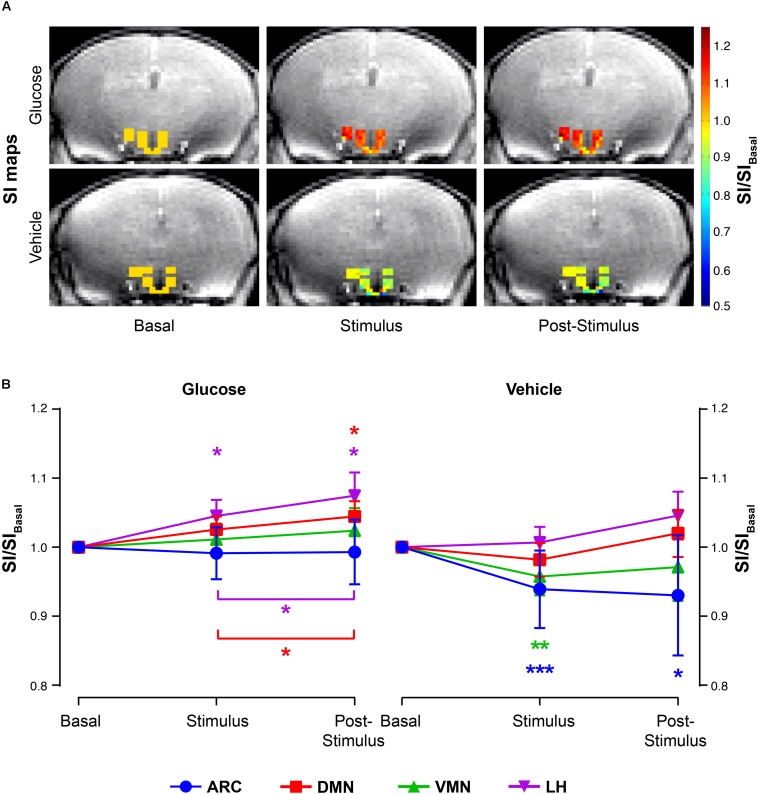
T_2_^∗^W signal changes in the hypothalamic nuclei after the administration of glucose or vehicle to fasting mice. **(A)** Representative color maps of normalized-to-baseline T_2_^∗^W SI from representative glucose (top) and vehicle (bottom) mice, at *basal* conditions (left), immediately after *stimulus* (middle) or during the *post-stimulus* periods (right). Colored voxel values in the hypothalamus are superimposed to a cerebral T_2_^∗^W image across the same plane. **(B)** Mean SI/SI_basal_ values of all voxels (±SEM) from the glucose (left) and vehicle (right) cohorts during the *basal*, *stimulus* and *post-stimulus* periods in the ARC, DMN, VMN and LH areas (in blue, red, green and purple, respectively) (^∗^*p* < 0.05, ^∗∗^*p* < 0.005, ^∗∗∗^*p* < 0.001).

Glucose administration induced a significant increase on normalized-SIs in the DMN and LH nuclei at both the *stimulus* and *post-stimulus* phases, as noted by the shift to red in the representative SI maps (Figure 3A, top panels) or by the evolution of mean SI/SI_basal_ values after the administration (Figure 3B, left). Differently, vehicle administration alone induced a decrease on the normalized-SI values of T_2_^∗^W on the ARC and VMN areas during the *stimulus* phase, which decreased further during *post-stimulus* in the ARC, as illustrated in the corresponding illustrative SI maps (Figure 3A, bottom panels) and mean SI/SI_basal_ graph panels (Figure 3B, right).

### DfMRI

#### Monoexponential Fittings

Fitting of the data to the monoexponential model yielded a high number of voxels with a goodness of fit *r*^2^ > 0.8 in the three directions measured (Table 1).

**TABLE 1:** Number of sub-hypothalamic nuclei voxels for each method.

		**ARC**		**DMN**		**VMN**		**LH**	
				
		**V**	**Glc**	**V**	**Glc**	**V**	**Glc**	**V**	**Glc**
T_2_^∗^W	*Basal*	39	68	42	84	72	126	47	84
	*Stim*	39	68	42	84	72	126	47	84
	*P-Stim*	39	68	42	84	72	126	47	84
*ADC*	*Basal*	32/22/17	60/48/24	48/47/48	72/61/62	72/71/72	125/113/87	48/40/41	75/64/57
	*Stim*	34/24/12	52/31/29	48/48/48	61/60/60	72/71/69	114/90/106	48/48/41	71/59/56
	*P-Stim*	36/24/13	48/29/37	48/41/48	62/52/84	72/63/72	90/77/124	48/36/41	61/48/72
SDP	*Basal*	18/10/3	39/17/29	25/23/16	28/31/26	29/19/26	43/19/28	17/9/18	33/23/33
	*Stim*	12/5/2	30/5/26	13/14/7	16/12/15	12/11/12	25/8/16	14/9/16	33/16/33
	*P-Stim*	12/9/2	22/12/22	10/16/7	18/15/14	17/10/12	25/13/17	16/9/16	26/23/33
*f*^∗^/*D*^∗^	*Basal*	12/10/11	24/13/23	20/9/16	54/24/44	26/17/30	75/58/76	34/16/19	49/21/6
	*Stim*	15/11/6	22/15/18	24/11/18	50/29/36	28/20/26	84/52/78	39/18/28	52/33/35
	*P-Stim*	14/8/4	21/11/19	19/11/19	53/26/39	20/16/22	78/41/67	32/20/24	57/29/46

*Final number of voxels used for the T_2_^∗^W, DfMRI-*high b* monoexponetial (represented by ADC parameter), DfMRI-*high b* biexponential (SDP) and IVIM approaches (*f*^∗^ and *D*^∗^ coefficients) during the *basal*, *stimulus* (*Stim*) and *post-stimulus* (*P-Stim*) phases on corresponding hypothalamic nuclei (ARC, DMN, VMN and LH). Values are provided for either the vehicle (V) or glucose (Glc) solution-administered animals and for the three orthogonal directions L-R/A-P/H-F used, when appropriate.*

**FIGURE 4:**
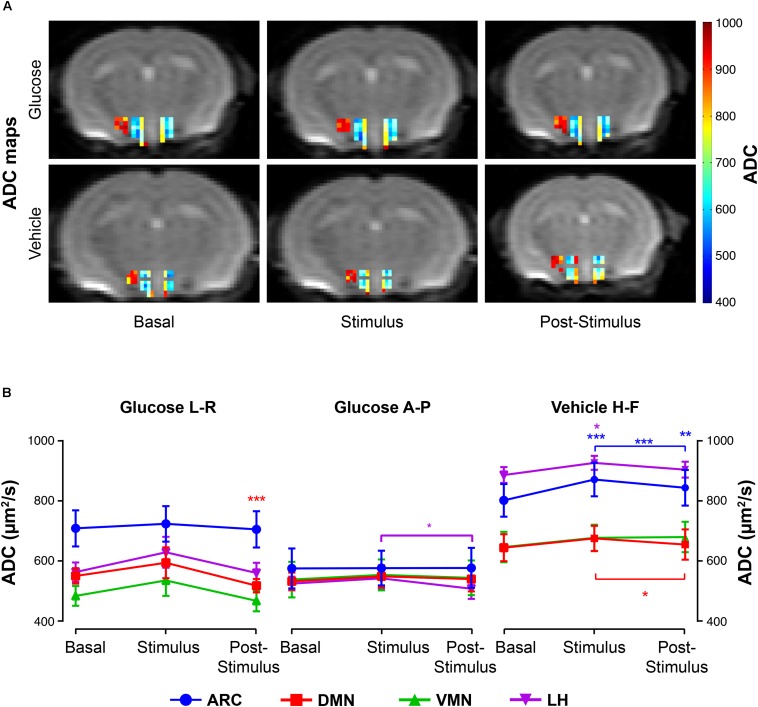
ADC changes in hypothalamic nuclei after the administration of glucose or vehicle to fasting mice. **(A)** ADC color maps (blue to red scale bar) of representative mice from the glucose (top) or vehicle (bottom) groups before its administration (*basal*), immediately after (*stimulus*), and during the corresponding *post-stimulus* periods (L-R direction). Colored coded hypothalamic voxels are shown superimposed to the corresponding DWI with *b* = 0. **(B)** Mean ADC voxel values (±SEM) in the *basal*, *stimulus* and *post-stimulus* periods in the ARC, DMN, VMN and LH nuclei (in blue, red, green and purple, respectively). Only ADC values with statistically significant differences between conditions) are represented (^∗^*p* < 0.05, ^∗∗^*p* < 0.005, ^∗∗∗^*p* < 0.001).

Glucose infusion decreased significantly ADC values in the DMN and LH nuclei during *post-stimulus*, as exemplified in the ADC maps (Figure 4A, top) and depicted in the graph boards (Figure 4B, left and center panels). Administration of the vehicle solution led to significantly increased coefficients in the ARC and LH at the *stimulus* period (Figure 4A, bottom) that decreased subsequently, remarkably in the ARC and DMN, with ADC values remaining higher than *baseline* only in the ARC area (Figure 4B, rightmost panel).

#### *High b* Biexponential Fittings

Fitting of the *high b* dataset to the biexponential model resulted in fewer voxels having a goodness of fit of *r*^2^ > 0.8, as compared to the monoexponential approach, especially during the *stimulus* and *post-stimulus* phases (Table 1). Nevertheless, the corresponding statistical analysis between SDP parameters revealed significant variations between states in the three orthogonal directions measured.

**FIGURE 5:**
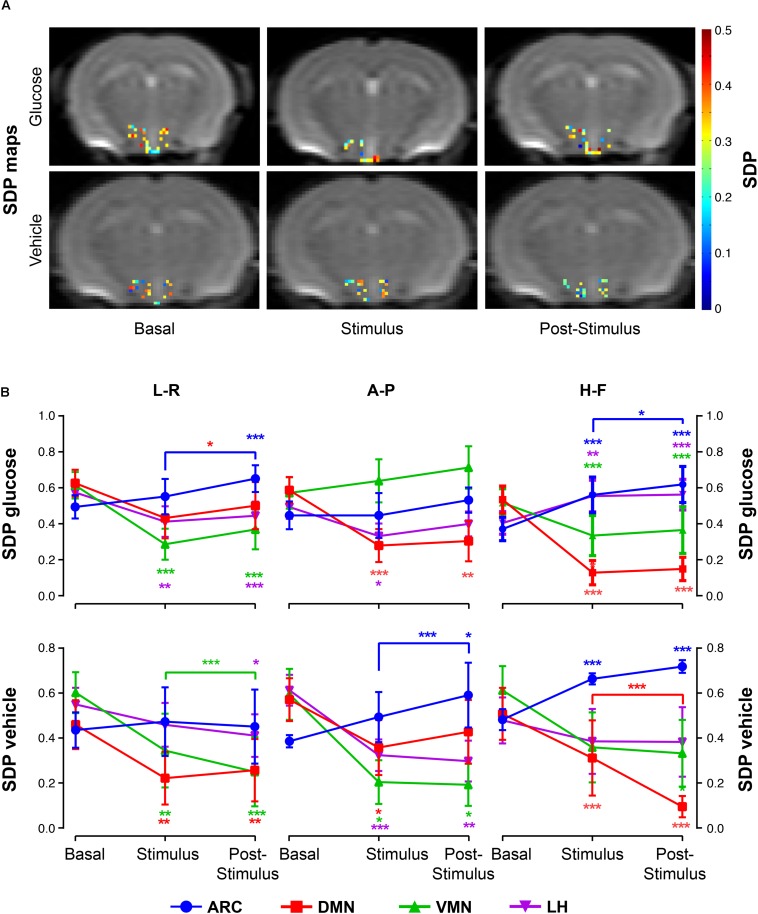
SDP variations in hypothalamic nuclei after the administration of glucose or vehicle to fasting mice. **(A)** Hypothalamic SDP maps of representative mice receiving glucose (top) or vehicle (bottom) administrations, in the *basal*, *stimulus* and *post-stimulus* conditions (L-R and H-F directions, respectively). **(B)** Line graphs of mean SDP voxel values (±SEM) from sub-hypothalamic nuclei of mice receiving glucose (top) or vehicle (bottom) administrations in the ARC, DMN, VMN and LH nuclei (in blue, red, green and purple, respectively) (^∗^*p* < 0.05, ^∗∗^*p* < 0.005, ^∗∗∗^*p* < 0.001).

The administration of glucose solution resulted in decreases of SDP values in the DMN and VMN areas during the *stimulus* and *post-stimulus* phases, but significantly increased values on the ARC, as depicted in the archetypical SDP maps (Figure 5A, top) and graph boards (Figure 5B, top). Additionally, the LH showed a directional-dependent behavior, with remarkable decreases along the L-R measurements and significant increases on the H-F direction (Figure 5B, top panels). Vehicle-only administrations induced generalized decreases of SDP values in all nuclei except on the ARC, where coefficients augmented with the stimulus (Figures 5A,B, bottom images and panels).

#### Intra Voxel Incoherent Motion Fittings

Analysis of the *low b* dataset using the IVIM approach yielded a relatively elevated number of voxels satisfying the condition *r*^2^ > 0.8, with more voxels having a high goodness of fit than the *high b* biexponential fitting (Table 1).

**FIGURE 6:**
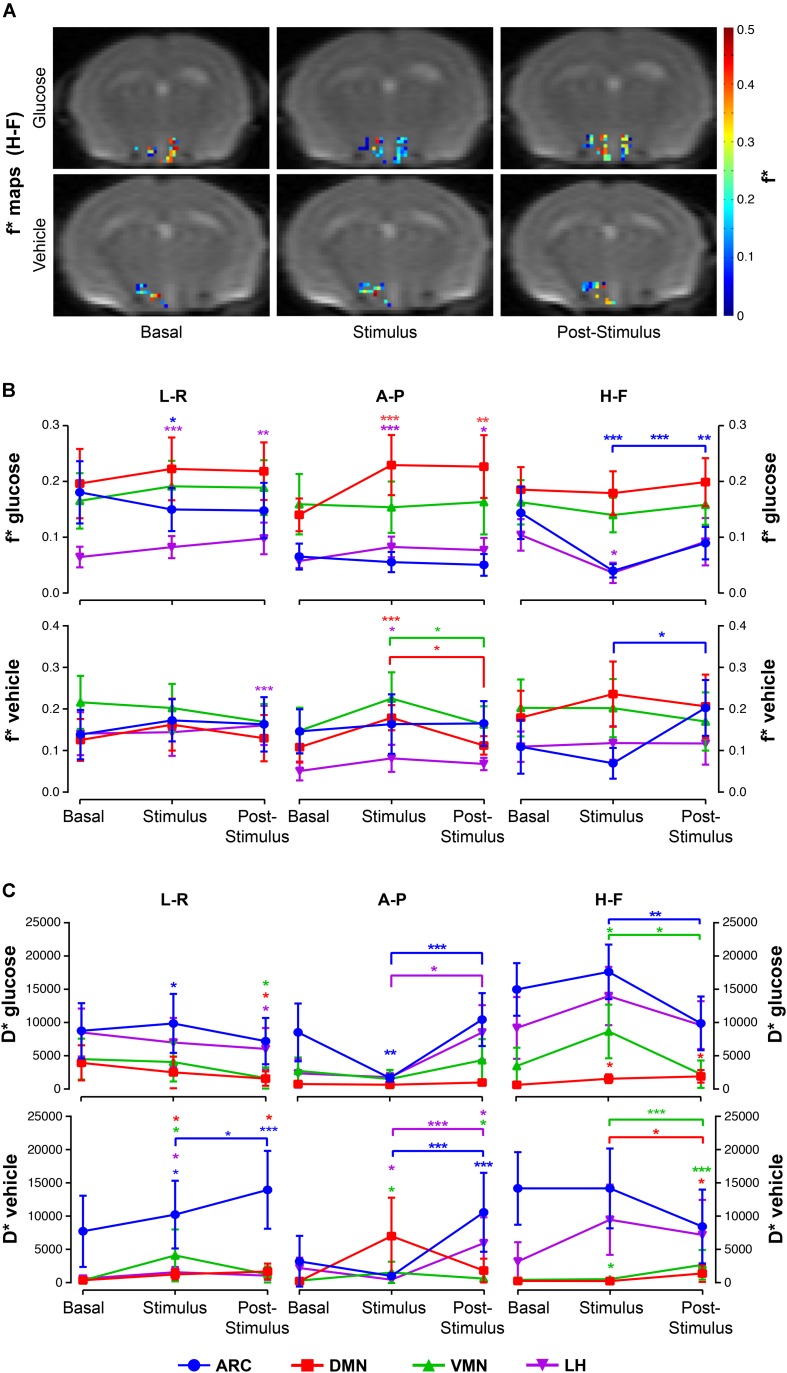
Intravoxel incoherent motion (IVIM) alterations in hypothalamic nuclei after the administration of glucose or vehicle to fasting mice. **(A)** Hypothalamic *f*^∗^ maps of representative mice receiving glucose (top) or vehicle (bottom) administrations, in the *basal*, *stimulus* and *post-stimulus* conditions. **(B)** Mean *f*^∗^ voxel values (±SEM) from sub-hypothalamic nuclei of mice receiving glucose (top) or glucose administrations (bottom), and **(C)** Mean *D*^∗^ (±SEM) coefficients of mice receiving the glucose solution (top) or vehicle (bottom) in the ARC, DMN, VMN and LH nuclei (in blue, red, green and purple, respectively) (^∗^*p* < 0.05, ^∗∗^*p* < 0.005, ^∗∗∗^*p* < 0.001).

Glucose administration resulted in increased *f*^∗^ values in the DMN at both *stimulus* and *post-stimulus* phases, but decreased *f*^∗^ fractions in the ARC during *stimulus* that augmented during the last phase (Figures 6A,B, top). The LH nuclei depicted directional-dependent variations, with glucose solution-induced increases in *f*^∗^ in the in-plane measurements and *f*^∗^ decreases along the H-F direction (Figure 6B, top). Results show that vehicle administration caused significant increases on the perfusion fraction *f*^∗^ in the DMN and VMN nuclei during *stimulus*, that decreased afterward, and significant increases during the *post-stimulus* measurements on the LH and ARC, as compared to *basal* and *stimulus* values, respectively (Figures 6A,B).

Glucose caused directional-dependent effects on the pseudo-diffusion coefficient *D*^∗^, with administration-induced increased coefficients at *stimulus* in the ARC, VMN and DMN that decreased significantly at the *post-stimulus* period (L-R and H-F directions), or decreased values during *stimulus* that recovered afterward in the ARC and LH (A-P measurements) (Figure 6C, top). Vehicle solution triggered *D*^∗^ increases in all areas during *stimulus* and *post-stimulus* phases (Figure 6C, bottom).

Generally, fitted values of both the perfusion fraction *f*^∗^ and pseudo-diffusion coefficient *D*^∗^ show high variability between animals and even voxels, with high standard deviations within each nuclei and condition (Figures 6B,C).

#### Immunohistochemistry

**FIGURE 7:**
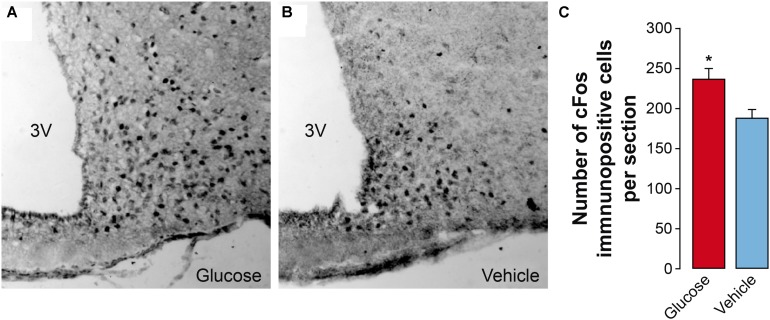
Effect of glucose administration on hypothalamic *c-fos* expression. **(A,B)** Representative immunohistochemical sections showing c-Fos immunoreactive cells in the arcuate nucleus (ARC) of mice treated with glucose **(A)** or saline (**B**, 0.9% NaCl). **(C)** Quantification of the number of c-Fos immunopositive cells in the arcuate nucleus obtained from sections similar to those shown in panels **(A,B)** (mean + SEM). A total of 8 sections per treatment were used. ^∗^*p* < 0.05, Student’s *t* test. 3V, third ventricle.

In order to obtain a correlation between the MRI parameters and more classical procedures of evaluation of hypothalamic function, we investigated *c-Fos* expression immunohistochemically 60 min after an i.p. injection of glucose. A significant number of *c-Fos* positive cells were detected in the ARC of vehicle-treated mice (Figure 7), consistent with the observed fasting-induced neuronal activation described in this hypothalamic location (Becskei et al., 2010; Wu et al., 2014). However, in glucose-treated animals, the number of *c-Fos* immunoreactive cells was significantly higher than in the vehicle-treated group. These results indicate that the changes observed by MRI imaging directly correlate with the occurrence of neuronal changes that coincide histologically in a spatial and temporal manner.

## Discussion

This work provides an integrated response-map of glucose-sensing and global energy metabolism of the mouse hypothalamus obtained using a combination of advanced fMRI implementations *in vivo* and immunohistochemical methods. We performed complete DWI and fMRI protocols, using T_2_^∗^W imaging, *high* and *low b* DfMRI and IVIM acquisitions, in fasting mice before, during, and following *i.p.* administrations of glucose or vehicle solutions, as well as *c-Fos* evaluation in the *post-stimulus* period. Additionally, the present study provides an exhaustive technical frame for the comparative evaluation of the different fMRI approaches implemented using the same paradigm. This allows, for the first time to our knowledge, the assessment of the similarities between the diffusion models considered by DfMRI, and the differences in microvascular perfusion information generated by T_2_^∗^W and IVIM methodologies.

### Hypothalamic Effects of Glucose Administration

Hormonal blood tests confirmed the expected endocrine responses (Ma et al., 2008), with the *post-stimulus* period being characterized by hyperglycemic and hyperleptinemic blood levels in the glucose group, as compared to the vehicle-only group.

Administration of glucose solutions to fasting animals induced local changes in the water molecules’ diffusion and microperfusion parameters, suggestive of increased cellular activity and blood flow, predominantly on the ARC, LH and DMN nuclei. More specifically, changes in diffusion were reflected by diminished ADC values in the DMN and LH, and increased SDP in the ARC. These effects are consistent with glucose-induced neurocellular activity in these areas. Diffusion parameters in the VMN and LH (L-R direction), and DMN (A-P direction) revealed reduced SDP values after the glucose stimulus, suggesting decreased cellular volumes and associated diminished activities. Indeed, decreases in ADC are thought to be associated to the cell swelling events concomitant to the increase in neuronal activity (Jin and Kim, 2008; Abe et al., 2017). SDP and FDP, on the other hand, have been proposed to represent, respectively, the fraction of water molecules diffusing in the vicinities of cell membranes –with slower motion- or the more distant components, (Le Bihan et al., 2006). Remarkably, the implementation of the biexponential approach to investigate brain activation has been used by different authors, yielding SDP values between 0.3 and 0.4 (Clark et al., 2002) that increase during activation, potentially reflecting an activation-induced cell swelling event (Lizarbe et al., 2013a). Compared to these previous studies, our results indicated slightly higher values in the fasting condition (0.4–0.6) that decreased after glucose administration (except in the ARC) reaching lower values (0.2–0.3). Notably, differences in the acquisition parameters, such as the range of *b*-values and diffusion times, may vary the capacity of detecting the slow diffusing molecules. Moreover, intrinsic properties of the tissue, namely brain regions, cell types or structures, or even –such could be our case- initial brain activity conditions, may account for the small differences reported between studies. Interestingly, independently of the diffusion model implemented, what has been consistently reported is an increase in the diffusion MRI signal that is concomitant to brain activation (Tsurugizawa et al., 2013; Nunes et al., 2019). Modifications in the microvasculature perfusion were characterized by remarkable SI rises in T_2_^∗^W in the DMN and LH, consistent with increases in blood oxygenation/microvascular flow (Kim and Ogawa, 2012).

Additionally, microperfusion variations were reflected by increased IVIM pseudo-diffusion fractions in the DMN and LH (in-plane measurements) but decreases in the ARC (H-F direction), while pseudo-diffusion coefficients had a variable response, suggesting a complex pattern of microvessel flow velocity changes. In summary, our study detected *in vivo* glucose-activated, but also glucose-deactivated, hypothalamic areas, in agreement with the described heterogeneous presence of glucose-excited or glucose-inhibited neurons in the diverse hypothalamic nuclei (Routh, 2010), and an additional –and concomitant- generalized increases of microvascular perfusion markers.

### Hypothalamic Effects of Vehicle Administration

Notably, the administration of the vehicle solution alone prompted unexpected hypothalamic responses involving alterations in neurocellular volumes and microvascular perfusion in different sub-hypothalamic nuclei. Cellular volume changes were revealed by generalized ADC increases following vehicle administration that dropped subsequently, and by post-infusion SDP decreases in all areas, except in the ARC, where values augmented significantly. These results point toward a cell shrinking effect of the saline solution administered as vehicle to fasting mice, detected mainly in the VMN, DMN and LH areas, revealing most probably reduced neuronal activity. In this sense, because it is known that animals diminish significantly water consumption during fasting (Jensen et al., 2013), and the hypothalamus contains neurons that regulate the control of thirst (Graebner et al., 2015), we hypothesize that the observed saline effects may be related to a local deactivation of thirst networks by the vehicle-only administration. Microvascular perfusion changes were revealed by T_2_^∗^W imaging analysis, disclosing relevant T_2_^∗^ SI decreases in the ARC and VMN areas, consistent with decreased microvascular flow/blood oxygenation levels, and with the aforementioned effects on the thirst balance mechanisms. However, IVIM measurements revealed increased pseudo-diffusion parameters in the DMN and LH immediately after the vehicle solution administration, and in the ARC to a lesser extent, reflecting a nuclei-dependent response to the saline (vehicle) bolus. Circumstances underlying the differences between T_2_^∗^W and IVIM imaging when addressing microvascular perfusion are discussed below.

### Comparison of the Different fMRI Approaches

In this work, we implemented four fMRI approaches, DfMRI with monoexponential fittings, DfMRI with biexponential fittings, T_2_^∗^W images and IVIM, to investigate the hypothalamic effects of the i.p. administration of glucose or vehicle solutions to fasting mice. Importantly, the first two methods provide information on neurocellular volume variations, and the last two on microvascular perfusion. Specific methodologically-inherent particularities should be considered, however, for an appropriate biophysical interpretation of the results.

The two DfMRI approaches applied used the same raw data to fit two diffusion models, described by the monoexponential and biexponential equations (Eqs 1 and 2), with the first model constraining in a single diffusion coefficient all diffusing water molecules (ADC), and the latter evidencing two diffusion phases (FDP and SDP), with their corresponding diffusion coefficients. Notably, our results revealed a strong agreement between changes in the resultant fitted parameters, with ADC increases generally paralleled by SDP decreases, and vice-versa. Biexponential fittings, however, lead to fewer good-fitting voxels but more nuclei showing statistically relevant changes between conditions, reflecting potentially higher specificity to detect cellular volume activation-induced changes.

The two microvascular perfusion-detection methods investigated depicted less correspondence between parameters, suggesting that the information provided by each methodology is not the same. Indeed, it is thought that the spatial origin of the activation spots detected by IVIM or T_2_^∗^W methods might differ, with IVIM being more sensible to flow in smaller vessels, and thus closer to activation sites (Le Bihan, 2019). Moreover, while IVIM accounts separately for the amount (*f*^∗^) and velocity (*D*^∗^) of circulating water molecules, and in a direction-dependent manner, the latter depicts mainly isotropic variations on the oxyhemoglobin/deoxyhemoglobin ratio, attributed to the changes in blood volume (Logothetis and Wandell, 2004), although the specific physiological origin of the T_2_^∗^W signal changes is complex (Gagnon et al., 2015). Finally, it should be noted that fitting of the low b datasets to the IVIM equations yielded pseudo-diffusion coefficients with very high standard deviation within neighboring voxels, potentially including contributions from water outside the microvascular network (Le Bihan, 2019). In this sense, we suspect that the differences reported between the T2^∗^ SI and IVIM parameters, soundly in the ARC, the closest area to the third ventricle, are related to this type of contaminations.

Additionally to the MRI techniques implemented, we used immunohistochemical methods to assess *ex vivo* the effects of the same glucose or vehicle solutions on fasting mice. We observed increased number of *c-Fos* immunoreactive cells in the ARC of glucose-treated animals, as compared to vehicle-only mice, indicating a higher glucose-induced *c-Fos* expression, and reflecting both higher neuronal and astrocytic activation response to the stimulus (Guillod-Maximin et al., 2004). These results are in agreement with the increased MRI-detected activity and perfusion-markers reported in the same area of glucose-treated mice, as compared to vehicle-only cohorts, thus providing histochemical support for the validation of the data obtained *in vivo*.

### Limitations

Anesthetics are known to affect neural and vascular physiology, and the type and dose chosen needs to be considered when comparing MRI studies of anesthetized mice (Masamoto et al., 2009). In particular, the use of isoflurane has been shown to alter the CBF response to forepaw stimulation in rats and to alter brain metabolism in mice (Boretius et al., 2013). In the DfMRI and T_2_^∗^W experiments described here, the three “states” investigated, are exposed to the same dosage of isoflurane anesthesia (1–1.5%), which is low compared to the cited studies. Accordingly, our results are expressed in relative terms –increase, decrease- between the different states. On these grounds, it is assumed that, even if the use of isoflurane anesthesia could potentially affect the brain response to glucose administration, as compared to the awaken mice; this effect should be limited and similar along the conditions investigated.

Both the IVIM and DfMRI sequences were acquired in three orthogonal directions, as a first attempt to detect potential differences in the orientation of the hypothalamic changes. Our results, however, did not show a clear preferential orientation of glucose or vehicle-induced effects. This could be related to fact that only three directions were assessed, while at least six non-collinear gradient orientations are needed to calculate the diffusion tensor, and thus the fractional anisotropy and axial and radial diffusivities (Le Bihan et al., 2001). In our study, the implementation of the (long) nine *b*-values sequences, designed to allow biexponential fittings, precluded the use of additional directions for time resolution restrictions. Future studies including acquisitions of at least six directions might investigate further orientation preferences of hypothalamic activation.

In this study, we reached a voxel resolution of 0.165 × 0.165 mm^2^ (in-plane) with 1.25 mm high. One of the goals of our investigation was to perform acquisitions at high *b*-values (up to 2000 s/mm^2^) to assess the slow components of water diffusion and implement biexponential fittings to the diffusion signal. Images acquired with high *b*-values, however, have low signal-to-noise ratio. In this sense, and to gain some signal, we opted to select relatively big voxel thickness (1.125 mm) but to maintain high in-plane resolution values. With this configuration, the main areas investigated (ARC, VMN, DMN, LH) remained inside the voxel limits. 1.25 mm is however, slightly bigger than the size mentioned nuclei, and the selection of thinner voxels could indeed result in the detection of subtle changes. Nevertheless, using our configuration, we managed to obtain information *in vivo* of the very small individual mouse sub-hypothalamic structures. There is no other technique, to our knowledge, capable of reaching such spatial resolution *in vivo*. Future studies, potentially reducing the number of *b*-values used, might investigate the effect of thinner selection of voxel sizes. A reduction of the number of *b*-values used would allow also better temporal resolution, but potentially preclude the implementation of biexponential approaches.

## Conclusion

In summary, the present study provides to our knowledge, the most robust array of non-invasive biomarkers reflecting hypothalamic neuronal activation or inhibition through its repercussions in water diffusion and T_2_^∗^ relaxation, validated with an independent histochemical biomarker. The present work brings the opportunity to evaluate the common response of these parameters to the glucose or vehicle administration paradigms and elaborate an integrative interpretation of the observed changes, helping to unravel the physiological origin of the DfMRI signal changes, a matter of debate in the last years (Miller et al., 2007; Bai et al., 2016; Williams et al., 2016; Nunes et al., 2019). Interestingly, our study shows also that even saline administration alone, triggers hypothalamic responses, delineating the extreme sensitivity of hypothalamic circuits to even small ionic perturbations.

Finally, the present study opens new avenues for further characterization of additional hypothalamic functions including energy metabolism, fluid and osmotic balance, thirst, behavior and their pathological implications as obesity or diabetes type 1 or 2, among others.

## Data Availability

The datasets generated for this study are available on request to the corresponding author.

## Ethics Statement

Animal Subjects: The animal study was reviewed and approved by ethical committee (Community of Madrid), the Spanish (R.D. 53/2013) and European Community (2010/62/UE) regulations.

## Author Contributions

BL designed the project, performed the MRI evaluations and data analysis, and wrote the first and final versions of the manuscript with all authors commenting. PL-L and SC designed the experiments and elaborated successive versions of the manuscript. AF-P and MV implemented the immunohistochemistry procedures, analysis, and discussion. VC and CL performed the blood tests analysis.

## Conflict of Interest Statement

The authors declare that the research was conducted in the absence of any commercial or financial relationships that could be construed as a potential conflict of interest.

## Footnotes

1 http://www.millipore.com

## Abbreviations

ADC: the apparent diffusion coefficient
ARC: arcuate nucleus
a.u.: arbitrary units
BOLD: blood oxygen level dependent
*D*_fast_: fast diffusion coefficient
DfMRI: diffusion-weighted functional magnetic resonance imaging
DMN: dorsomedial nucleus
*D*_slow_: slow diffusion coefficient
FDP: fast diffusion phase
GE: glucose excited neurons
GI: glucose inhibited neurons
IVIM: intravoxel incoherent motion
LH: lateral hypothalamus
PBS: phosphate buffered saline
SDP: slow diffusion phase
SNR: signal to noise ratio
VMN: ventromedial nucleus.
